# Personality Disorders and Attachment Trauma in Adolescent Patients with Psychiatric Disorders

**DOI:** 10.1007/s10802-023-01141-1

**Published:** 2023-10-27

**Authors:** Manuela Gander, Anna Buchheim, Kathrin Sevecke

**Affiliations:** 1 Institute of Psychology, University of Innsbruck, Universitätsstrasse 5-7, Innsbruck, 6020 Austria; 2 Present Address: Department of Child and Adolescent Psychiatry, Tirol Kliniken, Milserstrasse 10, 6060 Hall in Tirol, Tirol, Austria; 3 Department of Child and Adolescent Psychiatry, Medical University of Innsbruck, Innsbruck, 6020 Austria

**Keywords:** Personality Disorders, Childhood Trauma, Attachment, Gender, Mental Disorders, Adolescence

## Abstract

This study examined how personality disorders (PD) differ with respect to gender, attachment status and traumatic childhood experiences in adolescent psychiatric inpatients. In particular, we investigated attachment-related traumatic material underlying adolescent PD. Our sample consisted of 175 inpatient adolescents aged 14 to 18 years (77% female, *M*_*age*_ = 15.13, *SD* = 1.35; 23% male, *M*_*age*_ =14.85, *SD* = 1.41). Thirty-nine patients (22%) fulfilled the diagnostic criteria for a PD according to the *SCID-II PD*: 51% avoidant, 13% obsessive-compulsive, 13% antisocial, 19% borderline, 2% paranoid and 2% histrionic. In the total sample, eighty-three (47%) of our inpatients were classified with an unresolved attachment status using the Adult Attachment Projective Picture System (AAP). We did not find any significant gender differences for patients with and without a PD. Our results revealed a higher percentage of unresolved attachment status in patients with a PD. The in-depth analysis of the total sample showed that patients with a PD demonstrated more traumatic material in their attachment interviews indicating a greater severity of attachment trauma. Furthermore, patients with a PD reported higher scores on emotional and physical neglect. Intervention strategies targeting traumatic attachment-related themes might be useful to treat adolescents with PD.

## Introduction

Over the years, several studies suggest that the diagnostic features related to personality disorders (PD) are relatively stable in adolescent age groups (Chanen, 2022; Chanen & Thompson, 2018) and they support the idea that all PD types can also be identified in adolescents (Westen et al., 2014). Nevertheless, the stability of PD in younger age groups remains a contentious topic for debate (d’Huart et al., 2022). Morey and Hopwood (2013) outline that temporal stability of PD is considered as a complex notion and depends on clinical status and age range of the sample as critical factors in affecting the stability of PD over time. Existing findings, however, demonstrate that the stability of PD in adolescent age groups is found to be similar to the stability reported in adults (Grilo et al., 2001; Sharp & Wall, 2018).

Clinicians argue that an early detection and intervention in adolescent patients with PD is crucial for preventing adverse outcomes (Biberdzic et al., 2022). Evidence from a growing body of research shows that adolescent patients with a PD have elevated levels of psychopathological symptoms, a higher suicide risk, early onset-substance abuse, a lower quality of life, higher drop-out rates from education, an increased risk of being the victim or perpetrator of family violence and a poor physical and sexual health (Chanen et al., 2020). Furthermore, the presence of a PD is the fourth leading cause of disability among mental disorders, also in younger age groups < 18 (Mental Disorders Collaborators, 2022). Given that PD are among the most prevalent mental disorders in community samples with up to 10% (Winsper et al., 2020) and even rates up to 50% in clinical samples (Tyrer et al., 2015), they should be recognized as an important contributor to mental health and disease burden also in younger age groups. Several researchers emphasize that the economic burden is particularly high for patients with PD. This burden is much higher compared to help-seeking individuals with other mental disorders like anxiety disorders or major depression (Soeteman et al., 2008). Hastrup et al. (2019) highlight in their study that the annual total costs for patients with Borderline PD (BPD) were 16 times higher compared to matched controls without a BPD. The higher costs were observed particularly during the 5 years before they received a BPD diagnosis. Inpatient psychiatric treatment made up over 50% of the total costs. Moreover, spouses of patients with BPD demonstrated increased health care costs and significant productivity loss which present a further argument in favor of an early diagnosis and disorder-specific treatment.

While international classificatory systems might have expressed some caution to diagnose PD in patients below the age of 18 in the past, DSM-5 and ICD-11 have removed age-related caveats on the diagnosis of a PD based on the vast scientific evidence supporting an early diagnosis (Chanen, 2022). This vital reform to child and adolescent psychiatry and mental health sets the ground for further research on the pathogenesis, course and effective intervention for this group of young people who are at a high risk for adverse consequences in life. Studies in adolescent psychiatric samples are still rare.

Concerning the prevalence rate of PD in males and females, study results from adult samples are inconsistent. Some found a higher prevalence in males and described them as showing more externalizing symptoms, comorbid substance abuse and impulsive behaviors than females with PD (Amerio et al., 2023; Huang et al., 2009). Other researchers report higher levels of PD in females (Björkenstam et al., 2015) with the BPD as the most common one (Amerio et al., 2023). However, gender differences in adolescent PD have not yet been completely explored. Relevant literature in clinical and non-clinical settings is still scarce. For example, Mar et al. (2023) recently published a population-based study on the incidence of mental disorders and they report a higher incidence of PD in males particularly in the third decade of their life. In one of the only studies in hospitalized adolescent patients, females show higher rates of BPD, whereas males had higher rates of narcissistic PD but no gender differences were found for the other PD (Grilo et al., 1997). Although there are a handful of studies focusing on gender differences in the clinical presentation of BPD in adolescents (i.e. Bradley et al., 2005), prevalence rates regarding gender differences in adolescents with one or more PD in inpatients are still missing.

Furthermore, factors contributing to early manifestation of PD in adolescent psychiatric populations are still poorly understood. Childhood trauma and attachment are two important components that are considered to play a central role in the pathogenesis of PD.

Studies in adult psychiatric patients show that childhood maltreatment might be an important risk factor for the onset of personality pathology in adults (Buchheim et al., 2008; Kounou et al., 2013). In particular, individuals who experienced emotional neglect and abuse as well as physical abuse presented more severe PD symptoms compared to individuals without childhood trauma (Kounou et al., 2013; Wang et al., 2022). For adolescents, the majority of data is based on studies in younger patients with borderline features (Chanen & McCutcheon, 2013; Ibrahim et al., 2018; Sharp & Fonagy, 2015). This research suggests that all types of neglect and abuse were related to borderline features (Ibrahim et al., 2018) and more types of abuse were associated with increased levels of borderline features (Hecht et al., 2014) in younger populations. Nevertheless, traumatic childhood experiences are considered as non-specific risk factors for PD (Paris et al., 2014). Yet it is still unclear how these experiences might be related to adolescent PD. Furthermore, the majority of research is limited to the use of self-report measurements like the Childhood Trauma Questionnaire (CTQ, Bernstein & Fink, 1998) that retrospectively assess subjectively reported adverse childhood experiences. Although this measure is one of the most commonly-used and well-validated instruments in adolescent and adult populations, it has often been criticized because it is a retrospective self-assessment tool that includes the potential for recall bias (i.e. underreporting adverse childhood experiences due to feelings of shame and fear of stigmatization, depending on an individual’s memory performance and motivation to recall these experiences etc. (Wente et al., 2023). Given that PD are linked to enduring patterns of interpersonal functioning and emotion regulation (Hengartner et al., 2015; Nakash-Eisikovits et al., 2002) attachment theory might offer a conceptual bridge between these two components.

Concerning attachment patterns, associations between insecure-dismissing, insecure-preoccupied and unresolved attachment patterns and PD have been explored by research using various methodologies (Levy et al., 2015; Mikulincer & Shaver, 2016). Many studies on PD use attachment questionnaires evaluating an individual’s conscious thoughts about his or her attachment to significant others. This approach allows assessing attachment styles to a number of different caregivers (Roisman et al., 2007) and was used in several studies on adolescent personality pathology) and intervention studies, particularly in the field of dialectic behavioral therapy (DBT, (Bernheim et al., 2019) and mentalization-based therapy (MBT, (Cooper et al., 2021; Beck et al., 2016). However, questionnaires cannot assess unconscious aspects of attachment-related defenses and behaviors. Attachment interview procedures like the Adult Attachment Interview (*AAI*, (George et al., 1996) or the Adult Attachment Projective Picture System (*AAP*, (George & West, 2012) focus on mental representations of attachment by evaluating patterns of responses when participants talk about attachment situations. These interviews can classify attachment disorganization and trauma (George & Buchheim, 2014; George & West, 2012). The conceptual difference to childhood maltreatment assessed with a self-report questionnaire like the CTQ is that the disorganized-unresolved attachment pattern refers to the attachment disorganization and fear when confronted with attachment-related situations that represent a severe threat to the self (i.e. loss, separation, danger). In these situations, individuals need an attachment figure that provides comfort and safety. However, they experienced attachment figures that were unable to provide safety and help them to sooth their hyperarousal and thus they could not integrate traumatic attachment-related experiences. Hereby, the traumatic element is defined as the personal experience of a caregiver’s failure to respond sensitive to the needs of an individual during moments of severe attachment-related distress and threat which leads to feelings of helplessness, isolation and being caught in these experiences (Gander et al., 2018a; Zilberstein, 2014). Although some researchers refer to the “unresolved-disorganized attachment status” (i.e. van Hoof et al., 2019) in younger age groups, we use the term “unresolved attachment” which is commonly used in adolescent and adult age groups in the present study (Gander et al., 2021; Gander, Sevecke Gander et al., 2018a, b).

A large research base using narrative attachment measurements indicates that the unresolved classification predominates in adult patients with severe mental disorders, especially in those with PD (Beeney et al., 2017; Buchheim et al., 2017; Gander et al., 2018b; Levy, 2005; Nakash-Eisikovits et al., 2002). In the empirical literature on attachment much emphasis is given to adult patients with borderline PD (Buchheim & Diamond, 2018; Hengartner et al., 2015). Taken together, these studies demonstrate that an insecure and unresolved attachment quality is significantly linked with the severity of borderline symptoms in adolescence (Ibrahim et al., 2018; Sharp & Fonagy, 2015; Whalen et al., 2014). Additionally, studies have demonstrated a remarkable negative predictive value of a secure attachment pattern for any PD (Hengartner et al., 2015; Nakash-Eisikovits et al., 2002).

Despite robust evidence indicating a high prevalence of the unresolved attachment status in adults with PD, current research still lacks data on attachment representations in adolescents with a diagnosed PD. There are some recently published studies that focus on attachment and impaired personality functioning. In this context, childhood maltreatment was shown to represent an important risk factor for maladaptive personality functioning in adolescence. However, not all of them develop personality pathology (Paris et al., 2014). Luyten and Fonagy (2020) suggested that the unresolved attachment might play an important role for the link between childhood trauma, intrapersonal and interpersonal functioning. They propose that unresolved individuals with abusive or neglecting attachment figures might feel trapped in an approach-avoidance conflict. This conflict is expressed in the idealization-denigration cycles in their relation to other individuals. That is, they might perceive both the self and others as either extremely positive or as extremely negative depending on the situation. As a consequence they might feel confused regarding their self and the others - a characteristic that is commonly observed in traumatized adult patients with borderline PD (Luyten et al., 2020). Thus, traumatized individuals classified with an unresolved attachment pattern might have problems in developing a feeling of self-coherence and self-continuity as well as relatedness to others which are considered as core aspects of PD (Luyten et al., 2020). A study by Gander et al. (2020) could provide support for this conceptual bridge between attachment and trauma for the first time in younger age groups. They found that adolescents with a high amount of attachment trauma showed a lower resilience when facing traumatic childhood experiences resulting in greater impairments in personality functioning.

Even though research demonstrates important links between attachment experiences, childhood trauma and personality pathology, these results draw heavily from adult samples. Furthermore, many findings were based on patients with Borderline PD and did not include patients with other PD. As researchers progress towards creating an etiological model of adolescent PD, it is important to conduct research that incorporates adolescent patients who have been diagnosed with one or more PD. Although several research studies and the current classification systems DSM and ICD emphasize that most PD take their onset in adolescence and persist into adulthood, it is still unclear if patients with an adolescent onset differ from those with an onset in adulthood. There are a handful of studies comparing adolescent and adult samples with BPD suggesting that adolescents show similar neurobiological markers and interactions between temperament and adverse life events than those reported in adult samples with BPD (Paris, 2013). However, there is a lack of research on the role of trauma, particularly attachment-related trauma, in adolescents with PD.

In addition, attachment-related differences between adolescent inpatients with and without PD have not been addressed yet. In this context the Adult Attachment Projective Picture System (*AAP*, (George & West, 2012) has often been used as a viable tool to investigate the underpinnings of unresolved attachment in abnormal child development (Buchheim et al., 2008; Gander et al., 2020, 2021; Gander, Sevecke et al., 2018). This instrument allows for subjects to reveal traumatic material that indicates attachment-related danger or fear and has successfully been implemented in the field of adolescent psychiatry (Gander et al., 2020, 2021; Gander, Sevecke et al., 2018; Lenhart et al., 2022). Since PD are often discussed to be a consequence of disturbing attachment experiences, this approach might deepen our understanding of PD in youth.

### Aims of the Study

The present study analyses the prevalence rates of PD in adolescent psychiatric inpatients. We explore how the group of inpatients with a PD differ from inpatients without a PD with respect to gender, sociodemographic variables, attachment status, severity of attachment trauma and subjectively reported childhood trauma. This study expands upon previous research (Hengartner et al., 2015; Sharp et al., 2016) by adopting a recently developed approach to analyze attachment-related traumatic contents in adolescent patients with a diagnosed PD using an attachment interview. This approach helps us to illuminate different nuances of attachment-related trauma in patients with mental disorders. In contrast to earlier studies in the field (i.e. Sharp et al., 2016), our study does not exclusively focus on adolescents with BPD, but assesses all PD according to the DSM-IV-TR. Our study addresses the following research hypotheses: (1) Consistent with previous studies on prevalence rates of PD in clinical samples (Tyrer et al., 2015), we assume that more than 20% of our patients are diagnosed with one or more PD and that most of them have a comorbid axis I disorder (2) Although some researchers report higher prevalence rates of PD in females, particularly of the Borderline PD, in clinical settings (Tyrer et al., 2015), we expect no sex differences in the prevalence rate of PD in adolescents which is in line with the majority of research conducted in adult age groups (Gawda & Czubak, 2017). (3) We do not expect that patients with PD differ on sociodemographic variables (i.e. living situation, place of residence, number of siblings, marital status of the parents) from patients with no PD. However, given the higher drop-out rates from education/employment (Chanen, 2022), we expect to find a higher drop out of education or unemployment rate in our sample with PD. (4) As researchers have reported high rates of unresolved attachment status in adolescent and adult patients with Borderline PD (Hengartner et al., 2015), we assume that our patients with PD are more often classified as unresolved compared to patients without a PD. Finally, we suppose that clinical adolescents with PD show a greater severity of attachment trauma and subjectively traumatic childhood experiences.

## Materials and Methods

### Participants

Individuals were selected from a sample of 196 inpatients from the Department of Child and Adolescent Psychiatry at the Medical University of Innsbruck ranging in age between 14 and 18 years (150 females, *M*_*age*_= 15.09 years, *SD* = 1.36; 46 males, *M*_*age*_= 14.67 years, *SD* = 1.52) who participated in an ongoing study. We excluded all patients with an intelligence score < 85 assessed by the Hamburg Wechsler Intelligence Scale (Petermann & Petermann, 2008) (*n* = 4), an insufficient knowledge of the German language for the psychological assessments (*n* = 5) and incomplete questionnaires and/or psychological testing (*n* = 12). Our final inpatient sample for the analyses on attachment and PD was comprised of 175 adolescents (136 females, *M*_*age*_ = 15.13, *SD* = 1.35; 39 males, *M*_*age*_ = 14.85, *SD* = 1.41) aged 14 to 18. For the analyses on subjectively reported childhood trauma we had to exclude 9 further participants due to an incomplete or missing questionnaire (n = 166). We did not conduct missing data analysis in individuals who were excluded from the study. The present study was initially approved by the ethics committee of the Medical University of Innsbruck in 2016 and we received an extended approval in 2019 (No. 1120/2019). The study was carried out according to the declaration of Helsinki. Prior to inclusion in the study, we received written informed consent from all participants and their parents/legal guardians.

### Measures

#### Personality Disorders

Diagnoses were based on the results from the Structured Clinical Interview for DSM-IV (*SCID I*, Wittchen et al., 1997); *SCID II* (Fydrich et al., 1997). This is a semi-structured interview to assess the following DSM-IV Axis I disorders (*SCID-I*, German translation, see Wittchen et al., 1997): affective disorders, psychotic disorders, anxiety disorders, eating disorders, adjustment disorders, somatoform disorders and substance-related disorders. The *SCID-II* was used to make the 10 personality disorder diagnoses. We diagnosed categorical PDs according to the ICD-10 until December 2021 and replaced it with dimensional diagnoses of PD according to the ICD-11 in 2022. As the depressive and negativistic PDs were often considered as problematic due to their overlap with major depressive disorders and subsequently dropped from the DSM-IV-TR (Westen & Shedler, 1999), we decided to exclude these two from our analyses.

The interview procedure usually takes between one and two hours depending on the level of psychopathology of the interviewee. Although the interview is usually used in adults, it was administered successfully in adolescents as well (Wittchen et al., 1997). For our study, the complete *SCID* interview was conducted by trained clinical psychologists at the clinic. The reliability of the *SCID-I* and *II* interview for all the DSM diagnoses can be considered as good with Kappa values above 0.70 (Zanarini et al., 2000). In addition, numerous cross-national epidemiologic studies were done to demonstrate reliability and validity for these instruments in non-English-speaking samples and they show superior validity over other standard clinical interviews during the intake episode (First et al., 1997).We did not assess inter-rater reliability for the SCID-interview in the present study, which is also not reported in other research using the SCID interview (i.e. d’Huart et al., 2022).

#### Attachment Measurement

Attachment classifications and trauma-related material was assessed using the Adult Attachment Projective Picture System (*AAP*, George & West, 2012). This valid and reliable semi-structured interview consists of a set of pictures depicting one neutral stimulus (warm-up) and seven attachment-related stimuli representing themes of separation, death, solitude or fear (Bowlby, 1969). Individuals are required to tell a story about each picture by asking standardized questions: *What led up to that scene? What are the characters thinking or feeling? What might happen next?* The psychometric properties of the *AAP* have been tested in a number of studies in adults and adolescents demonstrating a good discriminant validity, inter-rater reliability, test-retest reliability (84% remained in the same attachment category after 3 months; κ = 0.78, *p* < .001) (Gander et al., 2017; George & Buchheim, 2014; George & West, 2012) and a high convergent validity with the *AAI* (94%, κ = 0.89 with a 95% narrow confidence interval [0.680, 1.100], *p* < .001 for the two group classifications (resolved-unresolved) (Buchheim et al., 2018; Gander et al., 2022). Based on different coding dimensions, the attachment narratives can be classified into four attachment categories: secure (*F*) narratives are characterized by thoughtful self-exploration and mutual enjoyment in attachment relationships, insecure-dismissing (*Ds*) narratives demonstrate a lot of distance in relationships (i.e. authoritarian orientation, normalization, social roles), insecure-preoccupied (*E*) narratives have a lot of insecurities and negative emotions like guilt, anger or shame and unresolved (*U*) narratives that describe an individual’s inability to seek protection from a caregiver or protect oneself when confronted with traumatic stressors like fear, isolation or death.

In the present study, we used a two-group coding dimension of resolved (F, Ds, E) and unresolved (U) attachment patterns. Furthermore, a new construct-based coding dimension allows to assess the severity of attachment trauma by counting the occurrence of the following unresolved trauma-related material in the attachment interview (≥ 0) (Buchheim et al., 2008; Gander et al., 2021; Gander, Sevecke et al., 2018): (1) danger/failed protection like abandonment or abuse, (2) helplessness like depictions of desperation or an emotional breakdown, (3) isolation like imprisonment or emptiness, (5) disturbing content like creepy or spooky elements, (6) obtrusions referring to the occurrence of unlicensed thoughts of threat or fear, (7) constrictions in which the individual is not able to complete the story due to overwhelming traumatic experiences.

In addition, differences on the occurrence of unresolved trauma-related material can be assessed in response to *alone AAP* pictures which portray individuals alone (i.e. bench – an individual sits alone on a bench, corner – a child stands askance in a corner) and *dyadic AAP* pictures showing individuals in attachment relationships (i.e. bed – a women and a child sit opposite each other on the child’s bed, ambulance – a child and a woman watch ambulance workers loading a stretcher into an ambulance car) (George & West, 2012). Alone picture stimuli require to evaluate whether story characters are able to preserve integrity (i.e. problem solving, repairing an attachment relationship) or make interpersonal connections with significant others when facing attachment-related traumatic situations. In dyadic pictures, coders assess mutual enjoyment, sensitive or functional care between the characters when confronted with stressful or even traumatic situations. This approach has not only demonstrated clinical utility in adult populations (Bernheim et al., 2022; Buchheim et al., 2008; Buchheim & George, 2011), but has also been successfully implied in adolescent psychiatric research (Gander et al., 2020, 2021; Gander, Sevecke et al., 2018) In accordance with many narrative-based attachment studies, half of our interviews (*n* = 87, 63.5%) were rated by two independent and reliable judges. Inter-rater reliability analysis revealed a kappa for the four-group classification of 96%, *κ* = 0.945 with a narrow 95% confidence interval [0.884, 1.006], *p* < .001. Concordance rate demonstrates an agreement in as many as 84 out of *n* = 87 cases. Disagreement between the judges was resolved by conference.

#### Childhood Trauma

We used the Childhood Trauma Questionnaire (CTQ, Bernstein & Fink, 1998) to evaluate traumatic childhood experiences (Klinitzke et al., 2012). This 28-item self-report questionnaire consists of the subscales emotional abuse and neglect, physical abuse and neglect and sexual abuse. On a 5-point Likert scale the individuals rate their childhood maltreatment from never true to very often true (scores 1–5, or from 5 − 1 for the 7 reverse-scored items). The CTQ allows the assessment of minimization or denial of childhood traumatic experiences from 0 (= never) to 1 (= all other responses). Scores on the global maltreatment scale range from 25 to 125 and for the subscales from 5 to 25, with higher scores indicating more severe maltreatment experiences. The psychometric properties of this instrument have been reported in several international studies and the questionnaire has demonstrated good construct validity and reliability. The German version of the CTQ has been tested in a large psychiatric (*n* = 363, (Bader et al., 2009) and community-based sample (*n* = 2000, (Klinitzke et al., 2012). It demonstrated an acceptable internal consistency (Cronbach’s alpha ≥ 0.80), construct validity (positive correlations with depression r = .36 (p < .001) and with anxiety r = .40, p < .001; negative correlations with life satisfaction, r = − .23, p < .001) (Klinitzke et al., 2012) and test-retest reliability suggesting that it may be considered as equivalent to the original English version (Bader et al., 2009). The internal consistency (Chronbach’s alpha) was very good in the present study (emotional abuse: α = 0.862, physical abuse: α = 0.838, sexual abuse: α = 0.933, emotional neglect: α = 0.851) except for physical neglect (α = 0.547) which is in line with several studies using the CTQ (Peng et al., 2023).

### Data Analysis

Statistical analyses were computed using IBM SPSS statistical software for Windows (version 25.0). Normal distribution of data and residuals were tested using the Kolmogorov-Smirnov test and Shapiro-Wilk test, respectively. We calculated descriptive statistics and group differences for the two PD groups (no PD and PD) using Pearson’s Chi-square tests (sex, marital status of parents, amounts of siblings, attachment classifications, diagnostic subgroups). The Kolmogorov-Smirnov test revealed that the continuous variables were normally distributed (p < .05). Therefore, we used the independent-samples t-test (two groups) to calculate group differences for attachment-related variables (N = 175) and subjectively reported childhood traumatic experiences (N = 166). We set the significance levels at α = 0.05 for all statistical analyses. We computed effect sizes by using Cohen’s conventions: small effect d = 0.2, medium effect d = 0.5 and large effect d = 0.8.

## Results

### Characteristics of the Study Sample

Based on the *SCID-I* interview, our patients were assigned to ICD-10 F- diagnoses: mental and behavioral disorders due to psychoactive substance use (F1, n = 3), schizophrenia, schizotypal, delusional, and other non-mood psychotic disorders (F2, n = 1), mood disorders (F3, n = 56), anxiety, dissociative, stress-related and somatoform mental disorders (F4, n = 38), eating disorders (F5, n = 47), pervasive and specific developmental disorders (F8, n = 1) and behavioral and emotional disorders with onset occurring in childhood and adolescence (F 9, n = 27). According to the *SCID-II* interview, 22.3% of our inpatient group fulfilled the criteria for a personality disorder (F6, n = 39). The majority of patients with a PD showed a comorbid axis I disorder and only two patients with a PD were not classified with a comorbid axis I diagnosis. In the PD group, 80% (n = 31) met the criteria for one PD and 20% (n = 8) for two PD. In total, the 39 patients fulfilled 47 PD diagnoses which are distributed as follows: 51% avoidant (n = 24), 13% obsessive-compulsive (n = 6), 13% antisocial (n = 6), 19% borderline (n = 9), 2% paranoid (n = 1) and 2% histrionic (n = 1).

Patients with PD and without PD did not differ on the following sociodemographic characteristics: living situation, place of residence, number of siblings, marital status of the parents, gender and age *t*(173) = 1.51, *p* = .133, *d* = 0.27. However, significantly more patients with a PD were unemployed/dropped out of school compared to those with no PD (Table 1).

**Table 1:** Sociodemographic characteristics between patients without PD and with PD

	Personality disorder				
	No PD (n = 136)	PD (n = 39)	χ ^2^	Φ	*p*
Living Situation (%)					
Living at home	86.7	82.1	0.550	0.056	0.458
Living alone/foster care	13.3	17.7			
Place of residence					
City	16.1	10.2	1.015	0.076	0.602
Town	22.1	20.5			
Village	61.8	69.3			
Amount of siblings					
Single child	27.2	15.4	2.285	0.114	0.319
One sibling	37.5	43.6			
Two or more siblings	35.5	41.0			
Marital status of parents			1.435	0.91	0.488
Married/partnership	51.5	41.0			
Single/divorced	43.4	51.3			
Deceased	5.1	7.7			
Occupation					
Attending school	81.6	71.8	6.791	0.197	0.034
Employed/trainee	13.2	10.3			
Unemployed	5.1	17.0			
Gender (%)					
Male	76.5	82.1	0.545	0.056	0.460
Female	23.5	17.9			

Abbreviations: *p* values refer to group differences based on χ^2^ tests; the classification for residence branches into the following three categories: City = population of 100,000–300,000; Town = population of 1,000–20,000, Village = population of < 1,000 people; *PD* personality disorder.

Concerning attachment classifications in the total sample, 4.6% of our adolescents had a secure (n = 8), 30.3% had an insecure-dismissing (n = 53), 17.7% had an insecure-preoccupied (n = 31) and 47.4% had an unresolved (n = 83) attachment status.

### Attachment Classifications in Patients with PD

In the total sample, descriptive analyses indicate a higher amount of unresolved attachment status in patients with a PD (61.5%, *n* = 24 vs. 38.5%, *n* = 15) than in those with no PD (43.4%, *n* = 59 vs. 56.6%, *n* = 77) *χ*^*2*^ (1, *n* = 175) = 4.01, *p* = .045, *Φ* = 0.15.

### Severity of Attachment Trauma in Patients with PD

As depicted in Table 2 our results demonstrate that patients with PD had a higher amount of traumatizing attachment-related material in their narratives (*M* = 1.26, *SD* = 1.35) than those without a PD (*M* = 0.77, *SD* = 1.13), *t*(173) = 2.26, *p* = .025, *d* = 0.39. In particular, an increased amount of traumatizing material occurred in pictures depicting elements of the caregiving relationship (adult-child dyadic stories) and elements of mutual enjoyment (adult-adult dyadic stories) in those with PD (*M* = 0.46, *SD* = 0.72) compared to those with no PD (*M* = 0.22, *SD* = 0.55), *t*(173) = 2.23, *p* = .027, *d* = 0.36 (see Table 2). Subsequently, we identified that the attachment-related theme of constriction (i.e. patients often freeze or are totally blocked from completing the attachment interview when confronted with interpersonal conflicts or failures) more often leads to attachment dysregulation in patients with PD, *t*(173) = 2.09, *p* = .037, *d* = 0.27.

**Table 2:** Traumatic attachment-related material and themes in the AAP stories of patients with and without PD

	Personality disorder						
	No PD (n = 136)		PD (n = 39)		*F*	*df*	*p*
	M	SD	M	SD			
Traumatic attachment-related material							
Alone AAP stories	0.65	1.48	0.80	0.89	0.028	173	0.575
Dyadic AAP stories	0.77	1.13	1.26	1.35	9.12	173	0.027
Total	0.77	1.13	1.26	1.35	3.295	173	0.025
Traumatic attachment themes							
Danger	1.24	3.09	2.05	4.22	2.049	173	0.184
Helplessness	0.47	1.26	0.56	1.02	0.000	173	0.671
Emptiness	0.25	1.00	0.39	0.99	1.154	173	0.459
Disturbing content	0.04	0.03	0.03	0.16	0.574	173	0.708
Spectral	0.16	0.56	0.26	0.89	3.146	173	0.407
Obtrusion	0.00	0.00	0.03	0.16	14.928	173	0.062
Constriction	0.01	0.09	0.08	0.35	18.457	173	0.037

Abbreviations: *PD* = personality disorder, *p* ≤ .05; *AAP* = Adult Attachment Projective Picture System

### Severity of Subjectively Reported Childhood Trauma in Patients with PD

Our results show that both clinical groups reported mean scores on the subscales emotional abuse and emotional neglect on the moderate to severe range, whereas on the subscales physical neglect, physical abuse and sexual abuse they reported mean scores in the low to moderate range. Independent t-tests revealed that patients with a PD reported higher scores on emotional neglect *t*(164) = 2.05, *p* = .042, *d* = 0.35 and physical neglect, *t*(164) = 2.26, *p* = .025, *d* = 0.35 compared to patients with no PD. To test whether participants underreport maltreatment, the CTQ measure includes a minimization/ denial scale that consists of three items: (1) *There was nothing I wanted to change about my family*, (2) *I had the perfect childhood*, and (3) *I had the best family in the world*. Yet interestingly, our findings show that the group of patients without a PD scored higher on the minimization items than the group of patients with a PD. Patients without a PD reported significantly more often that they had the best family in the world, *t*(164) = 37.48, *p* = .015, *d* = 0.52, and that they had the perfect childhood, *t*(164) = 97.18, *p* = .001, *d* = 0.71 (Table 3).

**Table 3:** Subjectively reported childhood trauma in patients with and without PD

	Personality disorder						
	No PD (n = 128)		PD (n = 38)		*F*	*df*	*p*
	M	SD	M	SD			
Traumatic childhood experiences							
Emotional abuse	10.46	5.38	12.08	4.87	0.606	164	0.098
Physical abuse	6.45	3.24	6.5	3.57	0.103	164	0.929
Sexual abuse	6.28	3.44	6.66	4.81	1.768	164	0.592
Emotional neglect	11.13	5.05	13.0	4.57	0.189	164	0.042
Physical neglect	6.91	2.52	8.03	3.18	1.013	164	0.025
Total	41.22	15.95	46.26	16.98	0.015	164	0.093

Abbreviations: *PD* = personality disorder, *p* ≤ .05;

## Discussion

This study examined the prevalence of PD in an adolescent psychiatric inpatient sample and found that 22% of our adolescents fulfilled the criteria for one or more PD. We did not find any sex differences for PD. This is the first study demonstrating that adolescent patients with PD have a higher amount of unresolved attachment patterns. Our in-depth analysis revealed that they had more traumatic material in their attachment narratives indicating a greater severity of attachment trauma. In particular, the theme of constriction left them in a state of dysregulation. In the AAP constriction refers to a condition in which patients freeze or are totally blocked from completing the attachment interview because their attachment-related defenses and resources break down and attachment-related fears become too overwhelming. In patients with PD this more often occurred in response to dyadic picture stimuli that depict elements of the caregiving relationship. Furthermore, patients with PD demonstrated higher scores on subjectively reported emotional and physical neglect in their childhood. Our findings might have important implications for the treatment of young people with PD.

Our results on prevalence rates of PD in adolescent inpatients is higher than rates reported in adult community samples (Winsper et al., 2020) but lower than rates reported in some adult clinical samples (Tyrer et al., 2015). Plausible explanations for these findings are rooted in the heterogeneity across studies. That is, some traits and categories might not be equally valid in different countries, variations in behavioral norms can have an influence on the PD prevalence rate and possible impacts of culture, race and ethnicity on PD specifically are still poorly understood (Winsper et al., 2020). Furthermore, the breadth of definition is crucial in determining accurate prevalence rates (Beckwith et al., 2014). We used the *SCID-II* interview that assesses moderate to severe PD; however, it does not include mild forms of PD which might explain why we found a lower rate compared to other studies. A study by Newton-Howes et al. (2010) demonstrates that exclusion of PD not otherwise specified and mild forms let prevalence rates drop from 40 to 10% in clinical samples.

Concerning the subtypes, our results demonstrated a relatively high rate of avoidant PD in adolescents. One reason for this finding might be the high prevalence of depressive disorders and Anorexia Nervosa (restrictive type) in our adolescent inpatient sample. Despite the fact that studies in clinical samples report higher rates of borderline PD (Sharp & Fonagy, 2015), our results are in accordance with data in adolescent and adult samples showing a high proportion of avoidant PD particularly in Anorexia Nervosa and depression (Magallón-Neri et al., 2014; Rotella et al., 2016).

In regard to gender differences, research points out that PD are equally common in both males and females; however, in clinical groups some studies report higher proportions of women with PD probably due to a greater help-seeking behavior, particularly in those with Borderline PD (Björkenstam et al., 2015). Interestingly, we could not replicate this finding in younger populations. This finding is partly consistent with some studies conducted in adults with PD. However, more large-scale research is needed in adolescent psychiatric populations (Conway et al., 2017) before strong conclusions can be drawn regarding the prevalence rates and sex differences. Additionally, generalization on gender differences should be treated with caution as they might be limited to our sample characteristics.

Concerning attachment patterns, half of our sample was classified with an unresolved attachment status. Although the high rate of this attachment pattern was also found in other studies in adolescents (Gander et al., 2021) and adults (Bakermans-Kranenburg & van IJzendoorn, 2009), the study of Venta et al. (2014) found a relatively low prevalence rate of 17% of unresolved individuals in their inpatient adolescent sample. One reason for these discrepancies might be the heterogenous diagnoses in inpatient samples. Whereas almost 50% of the Venta et al. (2014) study were diagnosed with an obsessive-compulsive disorder and oppositional defiant disorder which might be associated with a lower number of unresolved classifications, our sample consisted of patients with severe eating disorders and major depression – disorders more commonly associated with higher rates of unresolved attachment patterns (Delvecchio et al., 2014; Gander, Sevecke et al., 2018).

A major finding of this study was that adolescent patients with a PD demonstrated a higher amount of the unresolved attachment status and a higher severity of attachment trauma. This result corroborates previous research, particularly in the field of adult borderline PD (BPD) (Nakash et al., 2021). BPD is characterized by difficulties in the concept of the self, affect regulation, self-harming behaviors and episodic aggression. These problems in individuals with BPD manifest most prominently in interpersonal contexts (Weinstein et al., 2014) and they clearly resemble the contradictory and disoriented behaviors observed in disorganized infants (Beeney et al., 2017). Attachment theorists have already proposed that the unresolved attachment status provides a fertile ground for the onset of personality pathology (Buchheim & Diamond, 2018; Buchheim et al., 2017; Levy et al., 2015; Weinstein et al., 2014). Unresolved states of mind are associated with an individual’s perception of being abandoned and alone due to the failed protection by attachment figures. This experience of persistent dysregulated fear leads to the perception of an inner world that is characterized by incoherence, helplessness, isolation and chaos and threatens an individual’s psychological safety and self-integrity (Gander, Sevecke et al., 2018; George & West, 2012). This might result in severe intra- and interpersonal difficulties that are consistent with the disturbances observed in PD (Levy et al., 2015). The higher amount of traumatic attachment-related material in the narratives of our adolescent patients with PD compared to those with no PD provides additional support for this theoretical link. Furthermore, our in-depth analysis revealed that adolescent patients with PD show a lot of traumatic material in stories depicting interpersonal relationships. They were more often totally blocked from telling the story which might be indicative of their failure to contain the breakthrough of painful memories and attachment-related fears (George & West, 2011). In the story-telling process this becomes evident when individuals hand the picture back to the interviewer and make a statement that they are not going to respond to the stimulus or continue the story (i.e. *I feel something coming, I really don’t want to say something; it is the kind of scene I refuse to live, I don’t want to verbalize on this; I don’t want to do this one, that is enough*) (George & West, 2012). These findings indicate that interpersonal problems like impairments in empathy, reciprocity and attachment are at the core of personality dysfunction not only in adults (Hengartner et al., 2015) but in adolescents as well. In this context, our study retains a dimensional assessment of attachment by assessing the amount of traumatic material related to attachment. This is considered as a useful new approach in exploring attachment-related issues. Other authors who use the Child Attachment Interview use the dimensional scale of coherence (i.e. fresh speech, consistency, reflectiveness, comprehensibility, narrative production etc.) as an index of attachment disorganization (Sharp et al., 2016). However, the AAP does not assess coherence of the narratives so that the approach that is opted by Sharp et al. (2016) could not be applied to our study design. Furthermore, although Sharp et al. (2016) used the coherence of the narrative as an index of attachment disorganization, high scores on this scale are considered to be indicative of a lot of idealization and anger – typical features related to the insecure-dismissing and the insecure-preoccupied type (George & West, 2011). Thus, low coherence scores might not automatically be related to severe disorganization but can also be a characteristic of insecure but “resolved” individuals. Nevertheless, this approach might be an interesting direction for future research in adolescents with a diagnosed PD.

Concerning childhood maltreatment, patients with PD demonstrated moderate to severe scores on emotional abuse and neglect and moderate scores on the other childhood maltreatment experiences. These findings are in line with current findings demonstrating a link between traumatic experiences in childhood and personality pathology (Wang et al., 2022; Wong et al., 2022). In patients with BPD, researchers found that an exposure to different types of childhood trauma (i.e. emotional neglect and abuse, sexual and physical abuse) is associated with an increased risk for borderline symptoms in late childhood and adolescence (Ibrahim et al., 2018; Luyten, 2017). Even though numerous cohort studies suggest a strong link between childhood maltreatment and a wide range of adverse psychological symptoms (Wang et al., 2022), our results demonstrate that adolescent inpatients with PD report higher scores on emotional and physical neglect compared to those with no PD. In this context, attachment theory might offer a comprehensive view on underlying mechanisms that put adolescents at risk for an early onset of PD in response to these experiences. Researchers found that these types of trauma typically occur in the context of familial neglect and maltreatment (i.e. mental disorders in parents, poor parenting experiences) (Luyten, 2017). Given that the social proximity and prompt responsiveness of attachment figures are important for affect regulation difficulties in those with a PD (Hengartner et al., 2015), affect regulation deficits might become apparent in the face of traumatic childhood experiences (Bizzi et al., 2015). Patients with an unresolved attachment status are often incapable of tolerating their inner mental states and successfully regulate intense emotional states in response to maltreatment because of their lack of resources that provide attachment security (George & West, 2012). Instead, they remain in a state of dysregulation characterized by feelings of being trapped, isolated and helpless. This might lead to severe impairments in the domains of personality functioning. Gander et al. (2020) undermine this assumption by showing that adolescents with an unresolved attachment status report a lack of emotional self-experience and stabilizing perspectives, inconsistent self-images, a lack of autonomy and severe interpersonal problems. Thus, the quality of attachment might be an important factor involved in the onset of personality pathology in adolescence, particularly for those experiencing severe emotional and physical neglect in their childhood.

Interestingly, our patients with PD demonstrated less minimization of childhood maltreatment. This might be explained by the high proportion of patients with severe Anorexia Nervosa in our study sample. Some studies could show that these patients reach higher scores on the minimization/denial scale in the CTQ compared to other clinical groups (Couturier & Lock, 2006; Gander, Sevecke et al., 2018). Researchers argue that the denial of interpersonal difficulties and ruptures in parent-child relationships might represent a form of self-protection so that patients with Anorexia Nervosa do not have to communicate their inner feelings towards others. On the other hand, patients with PD are more likely to report childhood maltreatment (Wang et al., 2022); however their poor insight into their emotions, thoughts and psychological processes related to their childhood trauma as well as their low levels of self-regulation capacities (Goodarzi et al., 2018) might be related to severe intra- and interpersonal impairments.

The findings of the present study have to be interpreted in the context of some limitations. First, our adolescent inpatient sample consists largely of middle-class subjects with a relatively homogenous educational level and limited cultural diversity. Even though these sociodemographic variables are not related to attachment classifications (Gander et al., 2017), our results should be replicated in adolescent samples with other sociodemographic backgrounds. Furthermore, we only included psychiatric inpatients that might demonstrate a higher symptom severity and higher comorbidity than outpatient samples and we had a higher proportion of females. This is not unusual in research conducted in inpatient psychiatric samples due to the gender gap in mental health service use as men are reported to make fewer visits to mental health specialists than women (Pattyn et al., 2015). However, future studies might do well to conduct attachment, PD and childhood maltreatment in in- and outpatient adolescent samples with similar numbers of males and females. Second, our findings are based on a single point in time which increases the likelihood of method bias. In particular, we did not evaluate traumatic childhood experiences across developmental periods. This approach would allow an in-depth perspective on the cumulative effect of childhood maltreatment on adolescent personality pathology (Hecht et al., 2014). In this regard, the majority of our sample was assessed before the COVID pandemic so our data might not be generalized to prevalence rates during the pandemic years. Furthermore, the physical neglect subscale demonstrated the lowest internal consistency among all subscales of the CTQ which is in line with many previously published studies (Peng et al., 2023). Therefore, researchers suggested to re-examine or modify the items of this subscale and combine the physical and emotional neglect into one neglect scale in future research studies. Third, we used the SCID II interview to assess personality disorders on a categorical level. In this regard, future research should conduct a detailed analysis on attachment-related aspects for each PD. However, in our sample 20% of patients fulfilled the criteria for more than one personality disorder (some even up to 4 PDs according to the SCID-II interview). Although attachment-related differences among the different PD categories might be interesting venue for future research, our data suggests that in clinical practice some adolescents might show pathological scores in more than one PD. Similar findings of this overlap of PD facets have been reported in previous samples in adults as well, particularly in those with severe PDs, and thus represents a major limitation of the categorical model of PD. As PDs might not be best understood in terms of unique and nonoverlapping categories, a dimensional model of PD is supported which focuses on impaired levels of personality functioning that seems to be common to all PD types. Evidence for this theoretical framework stems from a recently published study by Simon and Bach (2022). Their analysis in a large clinical adult sample demonstrate that ICD-10 categorical PDs can be organized according to the four levels of ICD-11 dimensional PDs. In other words, their findings suggest a conceptualization of familiar PD categories in terms of ICD-11 PD severity and vice versa. Based on these results, creating one category for all PDs might represent an interesting approach as they all share impairments in the levels of personality functioning. Nevertheless, our approach does not come without conceptual and methodological limitations that need to be studied in future research.

Latest research in the field highlights the importance of using a dimensional approach of PD. Therefore, future research should additionally use validated measures that allow assessing DSM 5 PD on a dimensional level (Furnham et al., 2014). Although an alternative model for diagnosing PDs dimensionally has been integrated into the DSM-5 section III, the categorical definition of PDs has not been changed from the DSM-IV-TR. Thus, the categorical classification represents the main diagnostic guideline for all health care professionals in the U.S. including child and adolescent psychiatrists. Due to the aforementioned overlap of symptoms in our sample, studying symptom counts in larger samples might be an interesting approach for future research. Our research team is currently conducting a study on attachment and the dimensional classification of PD to explore whether we can replicate these findings using the new classification criteria in adolescents.

Fourth, as our sample size for each diagnostic subgroup was modest in our adolescent sample, our data did not allow analyzing potential effects of comorbid symptoms on the various measures. Yet more research focusing on comorbid disorder-specific aspects related to childhood maltreatment and attachment in adolescent patients with PD is needed before conclusions can be drawn in this field. In this context, a dimensional approach on impaired personality functioning might be a good alternative to expand our understanding of comorbid PD in mental disorders.

A fifth limitation is that a differentiation between all 4 attachment groups would do more justice to the differences between patients with and without PDs. However, in our inpatient clinical setting the low number of secure individuals did not allow a statistic analysis of the four attachment patterns. Furthermore, our approach is in line with other research studies that used the two-group classification of attachment which is a common method to distinguish between those with and without attachment trauma, i.e. individuals who can regulate and adapt to attachment-related stressors vs. individuals who dysregulate or become overwhelmed by traumatic attachment-related experiences (Gander et al., 2021; Gander, Sevecke et al., 2018). For future studies, recruiting an outpatient sample that might demonstrate a higher amount of secure attachment patterns would be helpful to conduct analysis using the 4-group classification. In this regard, some of our results show a p-value close to 0.05. However, there are two important aspects to consider: (1) Even though our sample size is not too small, it is also not very large, (2) our sample consists of adolescent inpatients with severe mental disorders. Including samples with less severe psychopathology (i.e. outpatient samples, community-based samples) might have increased the effect sizes and demonstrated greater p-values. Therefore, future studies including a sample with less psychopathology would contribute significantly to our understanding of attachment-related trauma and PD in adolescents.

Our study stands out as unique from previous studies as we used a large clinical inpatient adolescent sample to explore attachment status, severity of attachment trauma and childhood maltreatment in those with PD. Our findings do not only provide strong evidence for a high prevalence of over 20% for PDs in psychiatric units for younger age groups, but also for the presence of severe attachment trauma and childhood maltreatment in regard to physical and emotional neglect in these patients.

For clinical practice, our findings emphasize the importance of assessing attachment status and attachment-related traumatic material in adolescents with PD. This might help these patients to understand how their history of childhood trauma impacts their personality functioning on the interpersonal level and their affect regulation (Gander et al., 2020; George & Buchheim, 2014). Attachment-based intervention strategies might be particularly useful for them to face individual aspects of their attachment trauma (Gander et al., 2018a; Kobak & Kerig, 2015) and to develop more adaptive emotion regulation strategies in interpersonal contexts. In this regard, attachment-based interventions like the CONNECT program (Moretti et al., 2015), the attachment-based family therapy (ABFT, Ewing et al., 2015) or the MBT that is based on attachment theory (Beck et al., 2017) might be particularly helpful for patients with PD as they foster parental sensitivity and reflective functioning, dyadic affect regulation and mutuality within the parent-teen relationship. As suicidal ideation and suicide risk are common in patients with severe PD (Chanen, 2022), attachment-based treatments might be a useful approach because they demonstrated to more effectively reduce this behavior in teens than treatment as usual (Ewing et al., 2015; Waraan et al., 2021). A handful of recently published studies report that therapists that extensively use the attachment-based family treatment did not use standard CBT intervention techniques and that higher adherence to ABFT was related to positive treatment outcomes (Diamond et al., 2021). Furthermore, ABFT demonstrated higher rates of clinical symptom recovery and treatment retention in patients with suicidal ideation, major depression, anxiety and sexual abuse (Diamond et al., 2021). The findings of the present study might also expand on the use of psychotherapeutic techniques with adolescents. For example, a single case presentation of a 16-year old girl with BPD demonstrated how the use of attachment-related information from the AAP provided an insight into the patient’s traumatic dysregulation and helped to develop targeted therapeutic interventions supporting the patient to understand the emotional reactions to helplessness within an MBT setting (Gander & Sevecke, 2015). The integration of attachment issues in relation to unresolved attachment status and mentalization-based elements significantly improved the patient’s capacity to understand, interpret and differentiate between her own and other’s thoughts, feelings, desires and beliefs.

As empirically based treatments for PD in adults heavily rely on attachment theory (Buchheim & Diamond, 2018; Keefe et al., 2022; Levy et al., 2015), attachment-based treatment approaches hold the potential of a faster and greater symptom reduction, shortening the length of inpatient hospitalization and might yield better outcomes in regard to recidivism risk. The results of the present study might serve as a good foundation to optimize these therapeutic options for younger patients with personality pathology.

## Data Availability

The data that support the findings of this study are available on request from the corresponding author. The data are not publicly available due to privacy or ethical restrictions. Anna Buchheim: conceptualization, methodology, validation, reviewing and editing the manuscript. Kathrin Sevecke: conceptualization, methodology, validation, resources, reviewing and editing the manuscript, project administration.

## References

[CR1] Amerio, A., Natale, A., Gnecco, G. B., Lechiara, A., Verrina, E., Bianchi, D., & Aguglia, A. (2023). The role of gender in patients with borderline personality disorder: Differences related to hopelessness, alexithymia, coping strategies, and sensory profile. *Medicina (Kaunas)*, *59*(5), 10.3390/medicina59050950. 10.3390/medicina59050950 PMC10221694 37241182

[CR2] Bader K, Hänny C, Schäfer V, Neuckel A, Kuhl C (2009). Childhood trauma questionnaire - psychometric properties of a German version. Zeitschrift für Klinische Psychologie und Psychotherapie: Forschung Und Praxis.

[CR3] Bakermans-Kranenburg MJ, van IJzendoorn MH (2009). The first 10,000 adult attachment interviews: Distributions of adult attachment representations in clinical and non-clinical groups. Attachment & Human Development.

[CR4] Beck E, Bo S, Gondan M, Poulsen S, Pedersen L, Pedersen J, Simonsen E (2016). Mentalization-based treatment in groups for adolescents with borderline personality disorder (BPD) or subthreshold BPD versus treatment as usual (M-GAB): Study protocol for a randomized controlled trial. Trials.

[CR5] Beckwith H, Moran PF, Reilly J (2014). Personality disorder prevalence in psychiatric outpatients: A systematic literature review. Personal Ment Health.

[CR6] Beeney JE, Wright AGC, Stepp SD, Hallquist MN, Lazarus SA, Beeney JRS, Pilkonis PA (2017). Disorganized attachment and personality functioning in adults: A latent class analysis. Personal Disord.

[CR8] Bernheim D, Gander M, Keller F, Becker M, Lischke A, Mentel R, Buchheim A (2019). The role of attachment characteristics in dialectical behavior therapy for patients with borderline personality disorder. Clinical Psychology & Psychotherapy.

[CR7] Bernheim D, Buchheim A, Domin M, Mentel R, Lotze M (2022). Neural correlates of attachment representation in patients with borderline personality disorder using a personalized functional magnet resonance imaging task. Frontiers in Human Neuroscience.

[CR9] Bernstein, D. P., & Fink, L. (1998). *Childhood trauma questionnaire: A retrospective self-report -manual*. Psychological Cooperation.

[CR10] Biberdzic M, Grenyer BF, Normandin L, Ensink K, Clarkin JF (2022). A bifactor model of personality organization in adolescence: The validity of a brief screening measure assessing severity and core domains of functioning. Bmc Psychiatry.

[CR11] Bizzi F, Cavanna D, Castellano R, Pace CS (2015). Children’s mental representations with respect to caregivers and post-traumatic symptomatology in somatic symptom disorders and disruptive behavior disorders. Frontiers in Psychology.

[CR12] Björkenstam E, Björkenstam C, Holm H, Gerdin B, Ekselius L (2015). Excess cause-specific mortality in in-patient-treated individuals with personality disorder: 25-year nationwide population-based study. British Journal of Psychiatry.

[CR13] Bowlby, J. (1969). *Attachment and loss: 1. Attachment*. Hogarth Press.

[CR14] Bradley R, Conklin Z, Westen D (2005). The borderline personality diagnosis in adolescents: Gender differences and subtypes. Journal of Child Psychology and Psychiatry.

[CR15] Buchheim A, Diamond D (2018). Attachment and Borderline Personality Disorder. Psychiatric Clinics of North America.

[CR17] Buchheim, A., & George, C. (2011). Attachment disorganization in borderline personality disorder and anxiety disorder. In J. Solomon, & C. George (Eds.), *Disorganized attachment and caregiving* (pp. 343–382). Guilford Press.

[CR16] Buchheim A, Erk S, George C, Kächele H, Kircher T, Martius P, Walter H (2008). Neural correlates of attachment trauma in borderline personality disorder: A functional magnetic resonance imaging study. Psychiatry Research.

[CR18] Buchheim A, Hörz-Sagstetter S, Doering S, Rentrop M, Schuster P, Buchheim P, Fischer-Kern M (2017). Change of unresolved attachment in borderline personality disorder: RCT study of transference-focused psychotherapy. Psychotherapy and Psychosomatics.

[CR19] Buchheim, A., Labek, K., Taubner, S., Kessler, H., Pokorny, D., Kächele, H., & Karch, S. (2018). Modulation of gamma band activity and late positive potential in patients with chronic depression after psychodynamic psychotherapy. *Psychotherapy and Psychosomatics*, 1–3. 10.1159/000488090. 10.1159/000488090 29768272

[CR20] Chanen AM (2022). Debate: It is time to stop turning a blind eye to personality disorder in young people. Child Adolesc Ment Health.

[CR21] Chanen AM, McCutcheon L (2013). Prevention and early intervention for borderline personality disorder: Current status and recent evidence. British Journal of Psychiatry. Supplement.

[CR23] Chanen AM, Thompson KN (2018). Early intervention for personality disorder. Curr Opin Psychol.

[CR22] Chanen AM, Nicol K, Betts JK, Thompson KN (2020). Diagnosis and treatment of borderline personality disorder in young people. Curr Psychiatry Rep.

[CR24] Conway CC, Tackett JL, Skodol AE (2017). Are personality disorders assessed in young people?. American Journal of Psychiatry.

[CR25] Cooper EB, Anderson JL, Sharp C, Langley HA, Venta A (2021). Attachment, mentalization, and criterion B of the alternative DSM-5 model for personality disorders (AMPD). Borderline Personal Disord Emot Dysregul.

[CR26] Couturier JL, Lock J (2006). Denial and minimization in adolescents with Anorexia Nervosa. International Journal of Eating Disorders.

[CR27] d’Huart D, Steppan M, Seker S, Bürgin D, Boonmann C, Birkhölzer M, Schmeck K (2022). Prevalence and 10-year stability of personality disorders from adolescence to young adulthood in a high-risk sample. Frontiers in Psychiatry.

[CR28] Delvecchio E, Di Riso D, Salcuni S, Lis A, George C (2014). Anorexia and attachment: Dysregulated defense and pathological mourning. Frontiers in Psychology.

[CR29] Diamond G, Diamond GM, Levy S (2021). Attachment-based family therapy: Theory, clinical model, outcomes, and process research. Journal of Affective Disorders.

[CR30] Ewing ES, Diamond G, Levy S (2015). Attachment-based family therapy for depressed and suicidal adolescents: Theory, clinical model and empirical support. Attachment & Human Development.

[CR31] First, M. B., Gibbon, M., Spitzer, R. L., Williams, J. B. W., & Bejamin, L. S. (1997). *Structured clinical interview for DSM-IV axis II personality disorers (SCID II)*. American Psychiatric Press.

[CR32] Furnham A, Milner R, Akhtar R, De Fruyt F (2014). A review of the measures designed to assess DSM-5 personality disorders. Psychology.

[CR33] Fydrich, T., Renneberg, B., Schmitz, B., & Wittchen, H. U. (1997). *SKID-II. Strukturiertes Klinisches interview für DSM-IV. Achse II: Persönlichkeitsstörungen*. Hogrefe.

[CR39] Gander M, Sevecke K (2015). Bindungsbezogene Aspekte Bei Einer Jugendlichen Mit Einer Borderline-Persönlichkeitsstörung. Persönlichkeitsstörungen: Theorie Und Therapie.

[CR37] Gander M, George C, Pokorny D, Buchheim A (2017). Assessing attachment representations in adolescents: Discriminant validation of the adult attachment Projective Picture System. Child Psychiatry and Human Development.

[CR35] Gander, M., Diamond, D., Buchheim, A., & Sevecke, K. (2018a). Use of the adult attachment Projective Picture System in the formulation of a case of an adolescent refugee with PTSD. *Journal of Trauma & Dissociation: The Official Journal of the International Society for the Study of Dissociation (Issd)*, 1–24. 10.1080/15299732.2018.1451803. 10.1080/15299732.2018.1451803 29547072

[CR40] Gander M, Sevecke K, Buchheim A (2018). Disorder-specific attachment characteristics and experiences of childhood abuse and neglect in adolescents with Anorexia Nervosa and a major depressive episode. Clinical Psychology & Psychotherapy.

[CR34] Gander M, Buchheim A, Bock A, Steppan M, Sevecke K, Goth K (2020). Unresolved attachment mediates the relationship between childhood trauma and impaired personality functioning in adolescence. J Pers Disord.

[CR36] Gander M, Fuchs M, Franz N, Jahnke-Majorkovits AC, Buchheim A, Bock A, Sevecke K (2021). Non-suicidal self-injury and attachment trauma in adolescent inpatients with psychiatric disorders. Compr Psychiatry.

[CR38] Gander M, Karabatsiakis A, Nuderscher K, Bernheim D, Doyen-Waldecker C, Buchheim A (2022). Secure attachment representation in adolescence buffers heart-rate reactivity in response to attachment-related stressors. Frontiers in Human Neuroscience.

[CR41] Gawda B, Czubak K (2017). Prevalence of personality disorders in a general population among men and women. Psychological Reports.

[CR42] George, C., & Buchheim, A. (2014). Use of the Adult Attachment Projective Picture System in psychodynamic psychotherapy with a severely traumatized patient. *Frontiers in Psychology*, *5*. 10.3389/fpsyg.2014.00865 PMC4122203 25140164

[CR44] George C, West M (2011). The adult attachment Projective Picture System: Integrating attachment into clinical assessment. Journal of Personality Assessment.

[CR45] George, C., & West, M. L. (2012). *The adult attachment Projective Picture System: Attachment theory and assessment in adults*. Guilford Press.

[CR43] George, C., Kaplan, N., & Main, M. (1996). *The adult attachment interview*. University of California.

[CR46] Goodarzi N, Koomijani AH, Doniavi V (2018). Investigating the moderating role of self-knowledge processes and self-control in the relationship between childhood traumas and the severity of borderline personality traits. Annals of Medical and Health Sciences Research.

[CR48] Grilo CM, Walker ML, Becker DF, Edell WS, McGlashan TH (1997). Personality disorders in adolescents with major depression, substance use disorders, and coexisting major depression and substance use disorders. Journal of Consulting and Clinical Psychology.

[CR47] Grilo CM, Becker DF, Edell WS, McGlashan TH (2001). Stability and change of DSM-III-R personality disorder dimensions in adolescents followed up 2 years after psychiatric hospitalization. Compr Psychiatry.

[CR49] Hastrup LH, Jennum P, Ibsen R, Kjellberg J, Simonsen E (2019). Societal costs of borderline personality disorders: A matched-controlled nationwide study of patients and spouses. Acta Psychiatrica Scand.

[CR50] Hecht KF, Cicchetti D, Rogosch FA, Crick NR (2014). Borderline personality features in childhood: The role of subtype, developmental timing, and chronicity of child maltreatment. Development and Psychopathology.

[CR51] Hengartner MP, von Wyl A, Tanis T, Halmi W, Galynker I, Cohen LJ (2015). Severity of personality disorders and domains of general personality dysfunction related to attachment. Personal Ment Health.

[CR52] Huang Y, Kotov R, de Girolamo G, Preti A, Angermeyer M, Benjet C, Kessler RC (2009). DSM-IV personality disorders in the WHO World Mental Health Surveys. British Journal of Psychiatry.

[CR53] Ibrahim J, Cosgrave N, Woolgar M (2018). Childhood maltreatment and its link to borderline personality disorder features in children: A systematic review approach. Clinical Child Psychology and Psychiatry.

[CR54] Keefe JR, Levy KN, Sowislo JF, Diamond D, Doering S, Hörz-Sagstetter S, Clarkin JF (2022). Reflective functioning and its potential to moderate the efficacy of manualized psychodynamic therapies versus other treatments for borderline personality disorder. Journal of Consulting and Clinical Psychology.

[CR55] Klinitzke G, Romppel M, Häuser W, Brähler E, Glaesmer H (2012). The German version of the Childhood Trauma Questionnaire (CTQ): Psychometric characteristics in a representative sample of the general population. Psychotherapie, Psychosomatik, Medizinische Psychologie.

[CR56] Kobak RR, Kerig PK (2015). Introduction to the special issue: Attachment-based treatments for adolescents. Attachment & Human Development.

[CR57] Kounou KB, Bui E, Dassa KS, Hinton D, Fischer L, Djassoa G, Schmitt L (2013). Childhood trauma, personality disorders symptoms and current major depressive disorder in Togo. Social Psychiatry and Psychiatric Epidemiology.

[CR58] Lenhart L, Gander M, Steiger R, Dabkowska-Mika A, Mangesius S, Haid-Stecher N, Gizewski ER (2022). Attachment status is associated with grey matter recovery in adolescent Anorexia Nervosa: Findings from a longitudinal study. European Journal of Neuroscience.

[CR59] Levy KN (2005). The implications of attachment theory and research for understanding borderline personality disorder. Development and Psychopathology.

[CR60] Levy KN, Johnson BN, Clouthier TL, Scala W, Themes CM (2015). An attachment theoretical framework for personality disorders. Canadian Psychology.

[CR61] Luyten P (2017). Personality, psychopathology, and health through the lens of interpersonal relatedness and self-definition. Journal of the American Psychoanalytic Association.

[CR62] Luyten P, Campbell C, Fonagy P (2020). Borderline personality disorder, complex trauma, and problems with self and identity: A social-communicative approach. Journal of Personality.

[CR63] Magallón-Neri E, González E, Canalda G, Forns M, De La Fuente JE, Martínez E, Castro-Fornieles J (2014). Prevalence and severity of categorical and dimensional personality disorders in adolescents with eating disorders. European Eating Disorders Review: The Journal of the Eating Disorders Association.

[CR64] Mar J, Larrañaga I, Ibarrondo O, González-Pinto A, Las Hayas C, Fullaondo A, Consortium U (2023). Incidence of mental disorders in the general population aged 1–30 years disaggregated by gender and socioeconomic status. Social Psychiatry and Psychiatric Epidemiology.

[CR65] Mental Disorders Collaborators (2022). Global, regional, and national burden of 12 mental disorders in 204 countries and territories, 1990–2019: A systematic analysis for the global burden of Disease Study 2019. Lancet Psychiatry.

[CR66] Mikulincer, M., & Shaver, P. R. (2016). Adult attachment and emotion regulation. In J. Cassidy, & P. R. Shaver (Eds.), *Handbook of attachment: Theory, research, and clinical applications* (3 ed., pp. 507–533). Guilford Press.

[CR67] Moretti MM, Obsuth I, Craig SG, Bartolo T (2015). An attachment-based intervention for parents of adolescents at risk: Mechanisms of change. Attachment & Human Development.

[CR68] Morey LC, Hopwood CJ (2013). Stability and change in personality disorders. Annu Rev Clin Psychol.

[CR69] Nakash O, Nagar M, Razon L, Westen D (2021). Association between attachment patterns and personality disorders: A multimethod multi-informant study using a clinical sample. The Journal of Nervous and Mental Disease.

[CR70] Nakash-Eisikovits O, Dutra L, Westen D (2002). Relationship between attachment patterns and personality pathology in adolescents. Journal of the American Academy of Child and Adolescent Psychiatry.

[CR71] Newton-Howes G, Tyrer P, Anagnostakis K, Cooper S, Bowden-Jones O, Weaver T, team, C. s (2010). The prevalence of personality disorder, its comorbidity with mental state disorders, and its clinical significance in community mental health teams. Social Psychiatry and Psychiatric Epidemiology.

[CR72] Paris J (2013). Personality disorders begin in adolescence. J Can Acad Child Adolesc Psychiatry.

[CR73] Paris J, Perlin J, Laporte L, Fitzpatrick M, DeStefano J (2014). Exploring resilience and borderline personality disorder: A qualitative study of pairs of sisters. Personal Ment Health.

[CR74] Pattyn E, Verhaeghe M, Bracke P (2015). The gender gap in mental health service use. Social Psychiatry and Psychiatric Epidemiology.

[CR75] Peng C, Cheng J, Rong F, Wang Y, Yu Y (2023). Psychometric properties and normative data of the childhood trauma questionnaire-short form in Chinese adolescents. Frontiers in Psychology.

[CR76] Petermann, F. (2008). In U. Petermann (Ed.), *Hamburg-Wechsler-Intelligenztest für Kinder IV (HAWIK-IV)*. Huber.

[CR77] Roisman GI, Holland A, Fortuna K, Fraley RC, Clausell E, Clarke A (2007). The adult attachment interview and self-reports of attachment style: An empirical rapprochement. J Pers Soc Psychol.

[CR78] Rotella F, Fioravanti G, Ricca V (2016). Temperament and personality in eating disorders. Current Opinion in Psychiatry.

[CR79] Sharp C, Fonagy P (2015). Practitioner review: Borderline personality disorder in adolescence–recent conceptualization, intervention, and implications for clinical practice. Journal of Child Psychology and Psychiatry.

[CR81] Sharp C, Wall K (2018). Personality pathology grows up: Adolescence as a sensitive period. Curr Opin Psychol.

[CR80] Sharp C, Venta A, Vanwoerden S, Schramm A, Ha C, Newlin E, Fonagy P (2016). First empirical evaluation of the link between attachment, social cognition and borderline features in adolescents. Compr Psychiatry.

[CR82] Simon J, Bach B (2022). Organization of clinician-rated personality disorder types according to ICD-11 severity of personality dysfuntion. Psychodyn Psychiatry.

[CR83] Soeteman DI, Hakkaart-van Roijen L, Verheul R, Busschbach JJ (2008). The economic burden of personality disorders in mental health care. Journal of Clinical Psychiatry.

[CR84] Tyrer P, Reed GM, Crawford MJ (2015). Classification, assessment, prevalence, and effect of personality disorder. Lancet.

[CR85] van Hoof MJ, Riem M, Garrett A, Pannekoek N, van der Wee N, van IJzendoorn M, Vermeiren R (2019). Unresolved-disorganized attachment is associated with smaller hippocampus and increased functional connectivity beyond psychopathology. Journal of Traumatic Stress.

[CR86] Venta A, Shmueli-Goetz Y, Sharp C (2014). Assessing attachment in adolescence: A psychometric study of the child attachment interview. Psychological Assessment.

[CR87] Wang WL, Hung HY, Chung CH, Hsu JW, Huang KL, Chan YY, Chen MH (2022). Risk of personality disorders among childhood maltreatment victims: A nation-wide population-based study in Taiwan. Journal of Affective Disorders.

[CR88] Waraan L, Rognli EW, Czajkowski NO, Mehlum L, Aalberg M (2021). Efficacy of attachment-based family therapy compared to treatment as usual for suicidal ideation in adolescents with MDD. Clinical Child Psychology and Psychiatry.

[CR89] Weinstein L, Perez-Rodriguez MM, Siever L (2014). Personality disorders, attachment and psychodynamic psychotherapy. Psychopathology.

[CR90] Wente, V. M., Retz-Junginger, P., Crombach, A., Retz, W., & Barra, S. (2023). The suitability of the Childhood Trauma Questionnaire in criminal offender samples. *International Journal of Environmental Research and Public Health*, *20*(6), 10.3390/ijerph20065195. 10.3390/ijerph20065195 PMC10048956 36982104

[CR92] Westen D, Shedler J (1999). Revising and assessing axis II, part I: Developing a clinically and empirically valid assessment method. American Journal of Psychiatry.

[CR91] Westen D, DeFife JA, Malone JC, DiLallo J (2014). An empirically derived classification of adolescent personality disorders. Journal of the American Academy of Child and Adolescent Psychiatry.

[CR93] Whalen DJ, Scott LN, Jakubowski KP, McMakin DL, Hipwell AE, Silk JS, Stepp SD (2014). Affective behavior during mother-daughter conflict and borderline personality disorder severity across adolescence. Personal Disord.

[CR94] Winsper C, Bilgin A, Thompson A, Marwaha S, Chanen AM, Singh SP, Furtado V (2020). The prevalence of personality disorders in the community: A global systematic review and meta-analysis. British Journal of Psychiatry.

[CR95] Wittchen, H. U., Zaudig, M., & Fydrich, T. (1997). *SKID strukturiertes Klinisches interview für DSM-IV Achse I und II Handanweisung*. Hogrefe.

[CR96] Wong RS, Tung KTS, Ho FKW, Lee TMC, Chan KL, Bacon-Shone J, Ip P (2022). Associations between childhood maltreatment and psychiatric disorders: Analysis from electronic health records in Hong Kong. Transl Psychiatry.

[CR97] Zanarini MC, Skodol AE, Bender D, Dolan R, Sanislow C, Schaefer E, Gunderson JG (2000). The collaborative longitudinal personality disorders study: Reliability of axis I and II diagnoses. J Pers Disord.

[CR98] Zilberstein K (2014). Neurocognitive considerations in the treatment of attachment and complex trauma in children. Clinical Child Psychology and Psychiatry.

